# p62-dependent caspase-2 activation governs TDP-43 clearance and neuronal fate in ALS

**DOI:** 10.1038/s41419-025-08118-6

**Published:** 2025-11-06

**Authors:** Pavel I. Volik, Gelina S. Kopeina, Boris Zhivotovsky, Alexey V. Zamaraev

**Affiliations:** 1 Engelhardt Institute of Molecular Biology, Moscow, Russia; 2 Faculty of Medicine, MV Lomonosov Moscow State University, Moscow, Russia; 3 Division of Toxicology, Institute of Environmental Medicine, Karolinska Institutet, Stockholm, Sweden

**Keywords:** Cell death, Neurological disorders

Amyotrophic lateral sclerosis (ALS) is a fatal neurodegenerative disorder characterized by a loss of motor neurons. Progressive muscle weakness leads to the incremental development of respiratory failure and subsequent death of ALS patients 2–3 years after symptom onset. A central pathological hallmark of ALS is the formation of misfolded protein aggregates in degenerating motor neurons and surrounding oligodendrocytes [1]. However, the mechanism by which these cytoplasmic inclusions or aggregates affect neuronal function and cause motor neuron death remains poorly understood.

The majority of ALS cases are sporadic (sALS), while 5–10% are familial in origin (fALS). In 1993, Cu/Zn-binding superoxide dismutase (SOD1) was identified as the first protein aggregated in fALS due to the *SOD1* gene missense mutation. Since then, several novel ALS-associated gene defects have been identified, including mutations in *TARDBP*, the gene encoding TAR DNA-binding protein 43 kDa (TDP-43), a highly conserved DNA/RNA-binding protein that preferentially recognizes UG-rich and TG-rich motifs of RNA and DNA. TDP-43 has been reported as a prominent component of ubiquitinated and aberrantly phosphorylated cytosolic protein aggregates detected in 97% of all ALS patients, regardless of the mechanisms of disease onset. More than 50 mutations in *TARDBP* have been associated with ALS, confirming a crucial role of TDP-43 in ALS pathology [2]. N-terminally truncated forms of TDP-43, C-terminal fragments 35 kDa (CTF35) and 25 kDa (CTF25), are typically observed in ALS aggregations and associated with cell toxicity leading to the generation of insoluble ubiquitin- and phospho-positive cytoplasmic inclusions. TDP-43 CTFs could be generated by translation of alternatively spliced isoforms or proteolytic cleavage of full-length TDP-43. CTF35 and CTF25 are generated through proteolytic cleavage induced by caspase-3, -4 and -7, while calpains and δ-secretase can also cleave TDP-43, leading to CTFs’ formation. Other lower-abundance truncated fragments of TDP-43 (15–16 kDa, 22–25 kDa, and 33–37 kDa) have also been identified in ALS, but their role in disease pathology remains unclear [2].

Due to the cytotoxicity of TDP-43 species, the clearance of TDP-43 is a crucial cell survival mechanism. The degradation of full-length and CTFs of TDP-43 is normally mediated by the ubiquitin-proteasome system, which is disrupted by ALS-related mutations in the gene encoding Ubiquilin-2. Suppression of this clearance pathway stimulates a massive accumulation of TDP-43 aggregates in the cytoplasm of primary neurons [1]. Autophagy represents another critical clearance mechanism. The gain-of-function mutation (c.2155 A > G,p.M719V) in the deubiquitinase CYLD reduces autophagy, which correlates with TDP-43 mislocalization and aggregation [2]. Furthermore, TDP-43 aggregates, comprising full-length and CTFs of TDP-43, colocalize with autophagy-related protein sequestosome 1 (SQSTM1/p62). Previously, p62 was reported to decrease TDP-43 aggregation by promoting its autophagy- and proteasome-dependent degradation [3]. SQSTM1 overexpression has recently been found to promote CTF25 clearance in monkey substantia nigra [4]. Moreover, the identification of several pathogenic *SQSTM1* mutations in patients with ALS supports the involvement of p62 in TDP-43 clearance [5]. MicroRNA-183-5p, which suppresses *SQSTM1* expression, resulting in *TARDBP* upregulation and increased TDP-43 aggregation, can regulate the p62-dependent degradation of TDP-43 [6]. However, recent findings have indicated that p62 may play a role in the accumulation of toxic, misfolded fragments of TDP-43 in the cytoplasm. Nevertheless, the duration of experiments monitoring TDP-43 turnover in that study may provide a rationale for this controversial effect [7].

**Fig. 1:**
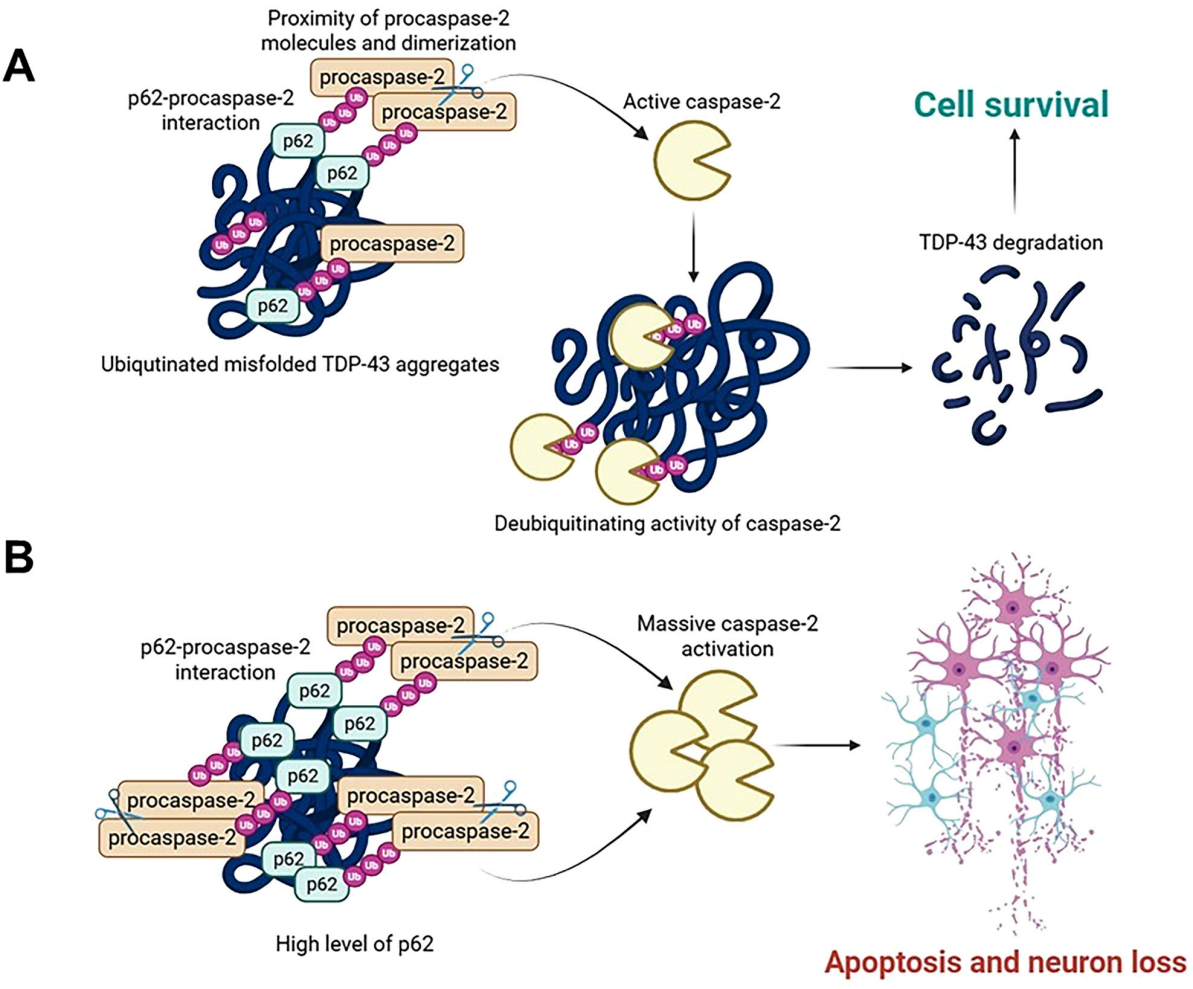
The mode of TDP-43 clearance by p62. **A** Activation of caspase-2 facilitates the removal of overloaded ubiquitin chains on TDP-43 prone to misfolding. **B** Caspase-2 switches to an apoptotic function in the presence of high p62 levels and excessive accumulation of TDP-43 inclusions.

In ALS, aggregates of TDP-43 are characterized by excessive accumulation of post-translational modifications, specifically phosphorylation, ubiquitination, and poly-ubiquitination [2]. A recent study [8] revealed that caspase-2, a multifunctional cysteine protease involved in cell death, genomic stability, and the oxidative stress response, could bind to poly-ubiquitinated conjugates via its allosteric ubiquitin-interacting motif-like region. It removes overloaded ubiquitin chains in a protease-dependent manner to mediate condensate disassembly and the degradation of misfolded proteins. The deficiency of *CASP2* results in the accumulation of stress-induced ubiquitinated complexes and pathological poly-ubiquitinated TDP-43 aggregates, promoting neuromuscular denervation in mice [9]. However, it is unclear how caspase-2 is activated in this scenario, since the enzyme is normally present in cells in the form of an inactive zymogen known as procaspase-2. The mechanism of caspase-2 activation is closely related to the induction of spatial proximity of procaspase-2 molecules, their dimerization, and subsequent autoproteolysis with active caspase-2 release. Recent findings unexpectedly demonstrate that caspase-2 can be similarly activated via ubiquitin-dependent interaction with p62 [10]. The abundance of p62 observed in ubiquitinated TDP-43-positive aggregates could promote proximity, dimerization, and autocleavage of procaspase-2 molecules, leading to the formation of active caspase-2, which can promote TDP-43 degradation, acting as a deubiquitinase. Thus, p62 could contribute to TDP-43 aggregates degradation through caspase-2 activation, preventing the development of ALS pathology (Fig. 1A).

Nevertheless, an important question related to the apoptotic function of caspase-2, activated by p62, remains. Under physiological conditions, the level of active caspase-2 molecules is sufficient to eliminate misfolded poly-ubiquitinated aggregates via deubiquitinase activity, but it is insufficient to induce neuronal apoptosis. However, in ALS pathology, excessive TDP-43 accumulation overwhelms these degradation pathways. The excessive accumulation of insoluble ubiquitinated p62-containing TDP-43 aggregates, whose clearance is counteracted, could promote intense caspase-2 activation through proximity-induced dimerization, switching its role from that of a proteostasis regulator to that of an apoptotic initiator, thereby eliminating neurons with irreversible proteostatic collapse (Fig. 1B**)**. The fact that *SQSTM1* overexpression accelerates ALS onset and shortens the lifespan of a mutant *SOD1*^*H46R*^-tg ALS mouse model supports this hypothesis [11]. Furthermore, a negative association of p62 levels with motor neuron loss in the spinal cord and disease duration was also found in patients with sporadic ALS [12]. These findings suggest dual context-dependent functions for caspase-2 in ALS and support further investigation of therapeutic strategies that modulate its activity to delay ALS progression.
